# Novel Insights into the Enigmatic Genetics of Male Breast Cancer in China

**DOI:** 10.3390/pathophysiology33010009

**Published:** 2026-01-20

**Authors:** Guan-Tian Lang, Xiao-Ling Weng, Yun Liu, Xin Hu, Zhi-Ming Shao, Zhen Hu

**Affiliations:** 1 Key Laboratory of Breast Cancer in Shanghai, Department of Breast Surgery, Precision Cancer Medicine Center, Fudan University Shanghai Cancer Center, Shanghai 200032, China; langguantian@126.com (G.-T.L.); xinhu@fudan.edu.cn (X.H.); zhi_ming_shao@163.com (Z.-M.S.); 2 Shanghai Key Laboratory of Systems Regulation and Clinical Translation for Cancer, State Key Laboratory of Systems Medicine for Cancer, Shanghai Cancer Institute, Shanghai 200127, China; wengxiaoling@renji.com (X.-L.W.); liuyun05050650@renji.com (Y.L.)

**Keywords:** male breast cancer, panel-based sequencing, whole-exome sequencing, *BRCA1/2*, *PALB2*

## Abstract

Objectives: The molecular characterization of male breast cancer (MaBC) has long been understudied, primarily due to its rare occurrence. Clinical management of MaBC remains profoundly challenging, with current therapeutic strategies largely extrapolated from female breast cancer protocols. Methods: Through panel-based sequencing targeting *BRCA1*, *BRCA2*, and *PALB2* variants, we delineated the genomic landscape of 96 MaBC cases. Subsequent whole-exome sequencing (WES) of 84 *BRCA1/2*- and *PALB2*-mutation-negative MaBC patients, compared against 4480 healthy controls, revealed compelling findings. Results: Pathogenic variants in *BRCA1/2* and *PALB2* were identified in 14.6% (14/96) of MaBC cases, with *BRCA2* mutations predominating at 12.5% (*n* = 12). Notably, one patient harbored the *BRCA1* c.4015G > T stop-gained mutation, while another exhibited the *PALB2* c.481_482dupGA alteration. Our analysis further uncovered 170 pathogenic/likely pathogenic mutations, with *RAD50*, *DMD*, *ARSA*, and *ABCC6* demonstrating recurrent mutations in MaBC. Conclusions: As the inaugural germline genomic investigation of MaBC in a Han Chinese population, this work reveals clinically actionable alterations with diagnostic and therapeutic implications. These discoveries not only advance our understanding of MaBC’s molecular architecture but also underscore the critical need for dedicated research into this malignancy.

## 1. Introduction

Male breast cancer (MaBC) represents a rare malignancy, constituting approximately 1% of all breast carcinomas and less than 1% of total cancer diagnoses in males [1,2]. Epidemiological data from 2016 estimated 2400 new cases and 440 breast cancer-associated mortalities among MaBC patients in the United States [3]. The limited availability of comprehensive clinical data—including patient demographics, tumor biology, therapeutic interventions, and prognostic outcomes—stems from the disease’s low incidence. Current clinical management predominantly relies on therapeutic paradigms extrapolated from female breast cancer studies, with insufficient characterization of MaBC-specific clinicopathological features. Furthermore, molecular profiling of MaBC remains inadequately explored. In this investigation, we performed panel-based sequencing analysis of *BRCA1*, *BRCA2*, and *PALB2* genes in 96 consecutive MaBC patients of Chinese Han ethnicity. Our primary objectives were to (1) determine the prevalence and spectrum of germline mutations in *BRCA1/2* and *PALB2* among Chinese male breast cancer patients and (2) conduct whole-exome sequencing (WES) to identify potential driver mutations underlying clinical manifestations in MaBC.

## 2. Materials and Methods

### 2.1. Study Population

**Figure 1:**
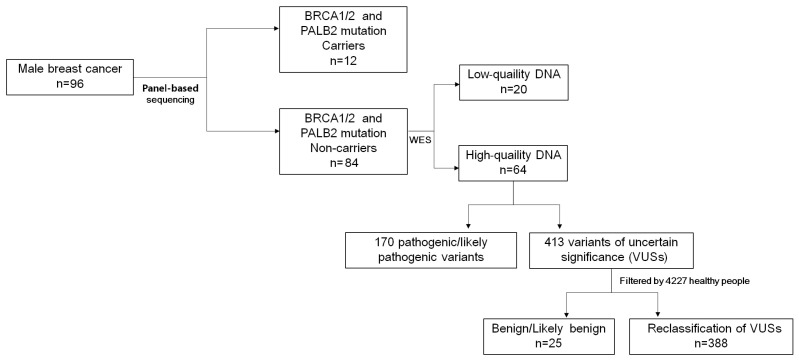
Study design and workflow.

A cohort comprising 96 MaBC patients was consecutively enrolled at Fudan University Shanghai Cancer Center (Shanghai, China) between January 2014 and November 2018. The study cohort comprised 4480 healthy control subjects obtained from a publicly available genetic database maintained by Zhejiang University School of Medicine (Hangzhou, China) [1,2,3]. Each participant signed a written informed consent form and ethical consent. This study received ethical approval from the Institutional Review Board of Fudan University Shanghai Cancer Center, with written informed consent obtained from all participants prior to sample collection and data acquisition. Clinically significant germline mutations were determined in our cohort of 96 unselected MaBC patients, according to Figure 1.

### 2.2. Targeted Sequencing

Peripheral blood specimens and comprehensive phenotypic data were systematically collected from all study participants. Genomic DNA extraction was performed using the VAZYME Blood Genomic DNA Kit (Vazyme Medical Technology, Nanjing, China) following the manufacturer’s protocols. The target-specific primers for the coding sequences of BRCA1 (NM_007300), BRCA2(NM_000059) and PALB2 (NM_024675) were designed using Primer3 as described previously [1]. The universal sequences (CS1: ACACTGACGACATGGTTCTACA; CS2: TACGGTAGCAGAGACTTGGTCT) were appended at the 5′-end of each left and right primer, respectively. Pre-amplification for tagged amplicon deep sequencing (TAm-Seq): The coding sequences of the two genes were amplified using a 6 µL PCR reaction mixture containing 3 µL of KAPA 2G Robust HotStart ReadyMix (2X) (Kapa Biosystems, Boston, MA, USA), 1 µL of primer mix with a 500 nM final concentration, and 2 µL of DNA template (10 ng/µL). The PCR conditions were as follows: initial denaturation for 2 min at 95 °C; 45 cycles of denaturation for 30 s at 95 °C, 30 s of annealing at 56 °C, and 1 min of extension at 72 °C; and a final extension step for 5 min at 72 °C. Following PCR amplification, 1.5 µL of Shrimp Aalkaline Phosphatase (SAP, Affymetrixx, Santa Clara, CA, USA)/Exonuclease I (Exo I, BioLabss, Ipswich, MA, USA) mix was added to 2.5 µL of PCR product and incubated for 60 min at 37 °C and then for 20 min at 80 °C. The SAP/Exo I mix contained 600 µL of SAP (1 U/µL), 240 µL of SAP buffer, 150 µL of Exo I (20,000 U/mL), 200 µL of Exo I buffer, and 3000 µL of ddH_2_O.

Sequencing adaptor and barcode primer addition were performed as described previously. The barcode primers (Fluidigmm Corporation, South San Francisco, CA, USA) consisted of the PE1 and PE2 sequences for Illumina cluster generation, a 10 bp barcode, and the CS1 and CS2 adaptors, used in pairs: PE1-CS1 with PE2-BC-CS2, and PE1-CS2 with PE2-BC-CS1. For each sample, 1 μL of the 100-fold-diluted PCR product was added to one of two PCR plates containing 9 μL of pre-sample mix containing 0.4 μL of 1 U/μL KAPA HiFi HotStart DNA Polymerase, 4 μL of 5× KAPA HiFi Buffer, 120 μM of each dNTP, and 4 μL of ddH_2_O. On the first plate, 10 μL of one primer pair containing an individual 10-base barcode (BC) sequence and tags for reading in one direction (PE1-BC-CS1 + PE2-CS2) was added to each well. On the second plate, 10 μL of primers containing (PE1-BC-CS2 + PE2-CS1) was added to each well. The corresponding wells on both plates contained primers with the same barcode sequence. Plated reaction products were amplified for 12 cycles: 95 °C for 10 min; 12 cycles of 95 °C for 15 s; 60 °C for 30 s; 72 °C for 3 min; and 1 cycle of 72 °C for 3 min.

For the DNA library, PCR products were barcoded and analyzed using gel electrophoresis to ensure that the expected insertion size was obtained. The products were then pooled together with an equal volume and purified using AMPure XP beads (Beckman Coulter, Indianapolis, IN, USA). The targeted DNA fragment was selected and extracted using E-Gel Precast Agarose Electrophoresis (ThermoFisher Scientific, Waltham, MA, USA) and the QIAquick Gel Extraction Kit (Qiagen, Santa Clara, CA, USA). The library was quantified by Agilent BioAnalyzer (Agilent, Santa Clara, CA, USA) and sequenced using the Illumina Xten platforms with paired-end reads of 150 bp as per the manufacturer’s instructions. Custom sequencing primers targeted to CS1 and CS2 targeted the paired reads and 10-base indexing (barcode) read as per the recommendations of Fluidigm.

### 2.3. Whole-Exome Sequencing

Briefly, 1 µg of DNA was sheared into short fragments (200–300 bp) using a Covaris S220 ultrasonicator (Covaris, Woburn, MA, USA). The DNA fragments were then end-repaired to generate adenylated 3′ ends. Adaptors with barcode sequences were then ligated to both ends of the fragments, and E-Gels were used to select DNA fragments of the targeted size. Next, 10 PCR cycles were performed, and the resulting product was purified. Whole-exome capture was performed using a TruSeq Exome Enrichment kit (Illumina, San Diego, CA, USA) according to the manufacturer’s protocol with slight modifications. After the Illumina sequencing libraries were amplified with 10 PCR cycles, capture probes were added, and the reaction mixtures were incubated at 65 °C for 24 h. The hybridized mixtures were then amplified with an additional 10 PCR cycles. The captured DNA libraries were sequenced with the Illumina HiSeq 2500 Genome Analyzer (Illumina, San Diego, CA, USA), yielding 200 (2 × 100) base pairs from the final library fragments.

### 2.4. NGS Data Processing and Variant Calling

Sequencing reads were aligned to the hg19 reference genome using Burrows-Wheeler Aligner (BWA, v0.7.13) [4], and the Genome Analysis Toolkit (GATK, v3.4) [5] was used for base quality score recalibration, indel realignment, and variant calling. We used GATK to filter the variants, with (i) QD < 2.0, (ii) MQ < 40.0, (iii) MQRankSum < −12.5, and (iv) ReadPosRankSum < −8.0 required. Variant functions were predicted using SnpEff (http://snpeff.sourceforge.net, accessed on 15 February 2018), PolyPhen-2 (http://genetics.bwh.harvard.edu/pph2/, accessed on 15 February 2018), PROVEAN (http://provean.jcvi.org/index.php, accessed on 15 February 2018), and SIFT (https://sift.bii.a-star.edu.sg/, accessed on 15 February 2018). Variant population frequency was annotated with ExAC (http://exac.broadinstitute.org, accessed on 15 February 2018), the 1000 Genomes database, and an internal database.

### 2.5. Variant Interpretation

In this study, only novel variants or variants with <1% population frequency in 1000 Genomes or ExAC were collected. The clinical significance of each variant was annotated according to the ACMG/AMP guidelines, using association results from this study, known clinical significance information from ClinVar (http://www.ncbi.nlm.nih.gov/clinvar/, accessed on 15 February 2018), computational data from in silico programs, and functional data. We manually inspected each variant using the Integrative Genomics Viewer to rule out false positives. After the annotation, the results were compared with classifications in ClinVar to identify additional information and determine the final classification of each variant, collapsed from a 5-tier to 3-tier classification system: pathogenic, benign, and uncertain significance. Variants classified to be pathogenic or likely pathogenic were considered pathogenic in this study. All pathogenic variants were validated by Sanger sequencing.

### 2.6. Statistical Analysis

Statistical comparisons of mutational status across clinicopathological characteristics in *BRCA* mutation carriers were conducted using Pearson’s chi-square test supplemented by Fisher’s exact test where appropriate. All statistical analyses were performed with SPSS Statistics software (Version 20.0; IBM Corporation, Armonk, NY, USA). A two-tailed alpha level of 0.05 was established a priori as the threshold for statistical significance throughout the study.

## 3. Results

### 3.1. BRCA1/2 and PALB2 Germline Mutations in Male Breast Cancer

Through targeted sequencing, our present investigation identified twelve deleterious germline mutations in *BRCA1/2* and *PALB2* genes among twelve of ninety-seven unselected MaBC patients, as detailed in Table 1. The observed prevalence rates exhibited marked heterogeneity in their distribution patterns: *BRCA1* mutations were identified in a single MaBC case, *BRCA2* mutations manifested in ten cases, and *PALB2* mutations were detected in a separate case. Molecular characterization revealed that the ten *BRCA2* mutations comprised six nonsense variants and four frameshift alterations. Notably, one patient exhibited a pathogenic *BRCA1* c.4015G > T stop-gained mutation, while another carried a *PALB2* c.481_482dupGA frameshift mutation confirmed as deleterious through clinical interpretation.

### 3.2. Frequent Germline Variants in Male Breast Cancer

**Figure 2:**
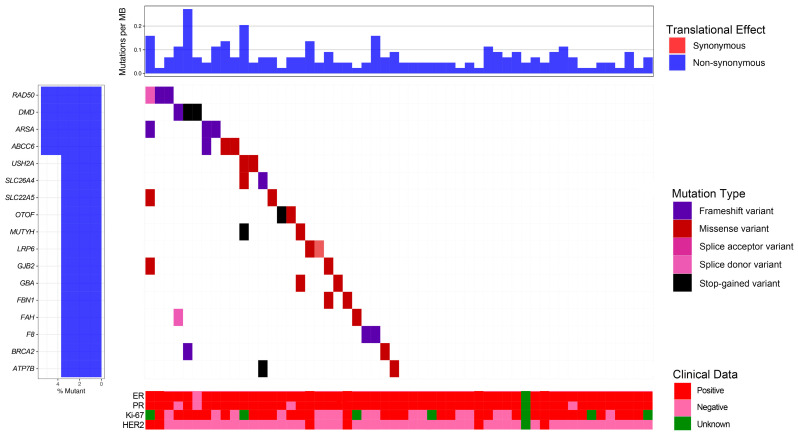
The spectrum of the pathogenic/likely pathogenic germline mutations by whole-exome sequencing analysis.

Subsequently, we employed whole-exome sequencing (WES) to investigate other frequent variants in the rest of 84 *BRCA1/2* non-mutated samples. Following quality control assessment, 20 patients were excluded due to insufficient DNA integrity and quantity (15 samples for low DNA amount, 4 samples for low sequencing depths, and 1 sample for pollution). Comprehensive analysis of WES data derived from the remaining 64 MaBC cases revealed 170 pathogenic variants (Figure 2 and Supplementary Table S1) alongside 388 variants of uncertain significance (VUSs) (Supplementary Table S2), with 25 VUSs excluded based on comparative analysis with control population data. Our WES analysis revealed two additional *BRCA2* mutations (c.5073delA and c.3387G > C) that were not detected in the targeted sequencing platform. Table 2 enumerates the four most prevalent pathogenic/likely pathogenic germline mutations identified, notably the *RAD50*, *DMD*, *ARSA*, and *ABCC6* genes, which were recurrently identified in three or more cases. These genetic aberrations potentially constitute impact on the oncogenesis and progression of MaBC. The mutational spectrum of the seven most frequently identified VUS-associated genes—*TTN*, *ATN1*, *ATXN3*, *SCN5A*, *DYSF*, *MYO7A*, and *POLE*—is exhaustively documented in Supplementary Table S2.

### 3.3. Associations Between MaBC Patients’ Characteristics and Mutation Status

We further explored the correlations between clinicopathological features of MaBCs and *BRCA1/2* or *PALB2* gene mutation status, with detailed results presented in Table 3. All identified mutations occurred in invasive breast carcinomas, including one case of encapsulated papillary carcinoma. All mutation carriers exhibited ER/PR-positive and HER2-negative molecular subtypes. Comparative analysis revealed potential differences in proliferative activity and tumor differentiation between mutation-positive and -negative groups. Specifically, the group with a *BRCA1/2* or *PALB2* mutation demonstrated a trend toward higher Ki-67 expression levels (85.7% vs. 59.8% with ≥15% positivity threshold; *p* = 0.061). Similarly, histological grading showed a higher proportion of grade 3 tumors in the mutation-positive cohort (42.9% vs. 18.3%; *p* = 0.077), though neither comparison reached statistical significance. Both groups demonstrated comparable distributions in tumor size (T classification), lymph node status, and TNM staging parameters.

## 4. Discussion

The number of cases of MaBC diagnosed is limited, and it has worse outcomes compared with female breast cancer [6]. Therefore, multi-institutional collaborative efforts will be essential to identify specific biomarkers for MaBC and further elucidate the molecular pathogenesis of this malignancy. Utilizing a hospital-based cohort, this investigation presents the first comprehensive genomic landscape of MaBC in the Chinese population. Our analysis revealed 14 deleterious germline mutations in the *BRCA1/2* and *PALB2* genes, along with 170 pathogenic/likely pathogenic variants and 388 VUSs. This study establishes foundational germline mutation profiles for MaBC, which constitutes a valuable reference for advancing mechanistic research and therapeutic development in this understudied disease entity.

Accurate interpretation of *BRCA1/2* variants is critical for risk assessment and precise treatment of breast cancer [7,8,9]. *PALB2* is an important DNA repair gene that is essential for its function in homologous recombination [10]. In the DNA damage response, PALB2 links BRCA1 and BRCA2, implementing the recombinational repair and checkpoint functions of BRCA2 in maintaining genome integrity [11]. This genomic profiling study systematically analyzed *BRCA1/2* and *PALB2* mutations in 96 consecutive MaBC cases without familial predisposition screening. The mutation spectrum revealed recurrent pathogenic variants in *BRCA2* (12.5%), significantly exceeding mutation rates observed in *BRCA1* (1.04%) and *PALB2* (1.04%). Targeted sequencing identified four exon 11 truncating mutations in *BRCA2* (p.Leu824 *, p.Glu2139 *, p.Leu1908fs, p.Gln1037 *), with whole-exome sequencing uncovering two additional exon 11 variants (p.Lys1691fs and p.Gln1129His), documented in Supplementary Table S1. Notably, two novel exon 22 mutations (p.Gln2960 * and p.Gln2941fs) were detected in functionally critical regions of BRCA2. These findings corroborate our prior research cohort (*n* = 46) showing 15.2% *BRCA2* mutation prevalence without *BRCA1* carriers. Comparatively, population-level data from UK MaBC cases demonstrates a 6% *BRCA2* mutation frequency, consistent with the established predominance of *BRCA2* over *BRCA1* alterations in male breast prediposition [12]. A US-based study examined 115 male breast cancer patients and identified 18 individuals carrying pathogenic *BRCA2* genetic mutations [13]. Our analysis demonstrates a statistically significant predominance of *BRCA2* mutations over *BRCA1* mutations in male breast cancer cohorts. Genetic profiling within our cohort identified a pathogenic *BRCA1* nonsense mutation (c.4015G > T, p.Glu1339Ter) in one patient and a deleterious *PALB2* frameshift variant (c.482_483delAG, p.Asp161GlyfsTer7) in another case. These findings align with previous research by Silvestri et al., who reported the recurrent *BRCA1* c.1984A > T (p.Lys662 *) nonsense mutation through targeted sequencing of *PALB2* in 48 *BRCA1/2*-negative MaBC specimens [14]. The mutagenic characteristics elucidated through our panel-based genomic profiling advance our understanding of the epidemiological distribution and mutational landscape of *BRCA1/2* and *PALB2* genes in MaBC.

No statistically significant associations were observed between mutation status and clinicopathological features. This lack of significance is likely attributable to limited statistical power, stemming from two main factors: the rarity of male breast cancer (which constitutes only about 1% of all breast cancer cases) and the low mutation-positive rate within our cohort. Nonetheless, potential trends in the data—such as a higher prevalence of invasive carcinoma (*p* = 0.061) and higher tumor grade (*p* = 0.077) in the mutation-positive group—may suggest a tendency toward more aggressive disease in this population. However, these observations should be interpreted with caution due to the small sample size, which restricts the generalizability and definitive interpretation of the findings.

The current study describes an in-depth exome analysis of the germline mutational landscape of MaBC. Among the significant MaBC-related genes identified in our study, *RAD50*, *DMD*, *ARSA* and *ABCC6* were detected multi-foci mutations. *RAD50*, a cancer susceptibility gene, encodes a component of MRN (Mre11-RAD50-Nbs1), which participates in DNA double-strand break repair and DNA-damage checkpoint activation [15,16,17,18]. *DMD* plays roles in numerous biochemical processes [19,20]. *ARSA* takes part in nervous system development [21,22]. *ABCC6* is involved in cardiovascular diseases [23,24]. The role of these deleterious mutations in MaBC progress remains to be defined.

In summary, this study constitutes a comprehensive exome-wide profiling of MaBC mutations. Our panel-based and whole-exome sequencing analyses delineated distinctive mutational characteristics specific to Chinese MaBC populations. These novel findings significantly advance our comprehension of mutational processes in MaBC pathogenesis through their genotoxic manifestations. The identification of recurrently mutated novel target genes provides critical insights into the evolutionary dynamics underlying complex mutational profiles during MaBC progression. Moreover, prioritizing the investigation of mutagenic mechanisms implicated in MaBC initiation should constitute a critical research focus. Systematic functional validation studies will be conducted to elucidate the potential utility of these genetic markers for MaBC risk assessment and early detection.

## Figures and Tables

**Table 1:** Pathogenic/likely pathogenic *BRCA1/2* and *PALB2* mutations identified in 12 male breast cancer cases.

Sample	Gene	Systematic Nomenclature	HGVS Protein Change	Annotation	Clinical Significance
1	*BRCA2*	c.8878C > T	p.Gln2960 *	Stop-gained	Pathogenic
2	*BRCA2*	c.2471T > G	p.Leu824 *	Stop-gained	Pathogenic
3	*BRCA2*	c.7558C > T	p.Arg2520 *	Stop-gained	Pathogenic
4	*BRCA2*	c.8172delG	p.Trp2725fs	Frameshift	Likely pathogenic
5	*BRCA2*	c.6415G > T	p.Glu2139 *	Stop-gained	Likely pathogenic
6	*BRCA2*	c.5722_5723delCT	p.Leu1908fs	Frameshift	Pathogenic
7	*PALB2*	c.481_482dupGA	p.Asp161fs	Frameshift	Likely pathogenic
8	*BRCA2*	c.37G > T	p.Glu13 *	Stop-gained	Pathogenic
9	*BRCA2*	c.1773_1776delTTAT	p.Ile591fs	Frameshift	Pathogenic
10	*BRCA2*	c.3109C > T	p.Gln1037 *	Stop-gained	Pathogenic
11	*BRCA2*	c.8820_8823delACAA	p.Gln2941fs	Frameshift	Likely pathogenic
12	*BRCA1*	c.4015G > T	p.Glu1339 *	Stop-gained	Pathogenic

* Terminal Codon.

**Table 2:** The top four pathogenic/likely pathogenic germline mutation-genes in 64 male breast cancer cases.

ID	Gene	Systematic Nomenclature	HGVS Protein Change	Annotation	Clinical Significance
ly6876	*RAD50*	c.2165delA	p.Lys722fs	Frameshift	Pathogenic
ly6853	*RAD50*	c.1245 + 2C > A		Splicing variant	Likely pathogenic
ly6762	*RAD50*	c.2165dupA	p.Glu723fs	Frameshift	Pathogenic
ly6825	*DMD*	c.4000G > T	p.Gly1334 *	Stop-gained	Pathogenic/Likely pathogenic
ly6885	*DMD*	c.5697delA	p.Lys1899fs	Frameshift	Pathogenic
ly6886	*DMD*	c.5530C > T	p.Arg1844 *	Stop-gained	Pathogenic
ly6820	*ARSA*	c.418delC	p.His140fs	Frameshift	Likely pathogenic
ly6870	*ARSA*	c.1492delC	p.Arg498fs	Frameshift	Likely pathogenic
ly6853	*ARSA*	c.302delG	p.Gly101fs	Frameshift	Pathogenic
ly6884	*ABCC6*	c.1990C > T	p.Pro664Ser	Missense	Pathogenic
ly6820	*ABCC6*	c.196dupT	p.Ser66fs	Frameshift	Pathogenic
ly6865	*ABCC6*	c.3412C > T	p.Arg1138Trp	Missense	Pathogenic

* Terminal Codon.

**Table 3:** Association of MaBC characteristics with *BRCA1/2* or *PALB2* mutation status.

Characteristics	No. of Patients	Mutation Status				*p*
		Non-Carriers (*N* = )		Carriers (*N* = )		
		No.	%	No.	%	
**Histologic classification**						
Carcinoma in situ	6	6	7.3%	0	0.0%	**0.061**
Invasive ductal carcinoma	67	54	65.9%	13	92.9%	
Other invasive carcinoma	23	22	26.8%	1	7.1%	
**ER status**						
Negative	3	3	3.7%	0	0.0%	**0.525**
Positive	92	78	95.1%	14	100.0%	
Unknown	1	1	1.2%	0	0.0%	
**PR status**						
Negative	8	8	9.8%	0	0.0%	**0.224**
Positive	87	73	89.0%	14	100.0%	
Unknown	1	1	1.2%	0	0.0%	
**HER2 status**						
Negative	88	74	90.2%	14	100.0%	**0.267**
Positive	6	6	7.3%	0	0.0%	
Unknown	2	2	2.4%	0	0.0%	
**Ki67 status**						
<15%	25	24	29.3%	1	7.1%	**0.112**
≥15%	61	49	59.8%	12	85.7%	
Unknown	10	9	11.0%	1	7.1%	
**Tumor size**						
≤2 cm	73	62	75.6%	11	78.6%	**0.556**
>2 cm	23	20	24.4%	3	21.4%	
**Tumor grade**						
I	2	2	2.4%	0	0.0%	**0.077**
II	46	39	47.6%	7	50.0%	
III	21	15	18.3%	6	42.9%	
Unknown	27	26	31.7%	1	7.1%	
**Cancer emboli**						
Negative	27	24	29.3%	3	21.4%	**0.301**
Positive	52	42	51.2%	10	71.4%	
Unknown	17	16	19.5%	1	7.1%	
**Lymph node status**						
Negative	68	57	69.5%	11	78.6%	**0.367**
Positive	28	25	30.5%	3	21.4%	
**Stage**						
0	6	6	7.3%	0	0.0%	**0.458**
I	49	41	50.0%	8	57.1%	
II	29	24	29.3%	5	35.7%	
III	12	11	13.4%	1	7.1%	

## Data Availability

The original contributions presented in this study are included in the article/Supplementary Materials. Further inquiries can be directed to the corresponding authors.

## Supplementary Materials

The following supporting information can be downloaded at https://www.mdpi.com/article/10.3390/pathophysiology33010009/s1. Table S1: The 170 pathogenic/likely pathogenic germline mutations in 64 male breast cancer cases. Table S2. The 413 VUS germline mutations in 64 male breast cancer cases.

## Abbreviations

The following abbreviations are used in this manuscript:

MaBC	Male breast cancer
BRCA	Breast Cancer Susceptibility Gene
WES	Whole-exome sequencing
VUS	Variant of uncertain significance
