# Supplementary material for: Novel Insights into the Enigmatic Genetics of Male Breast Cancer in China
Source: Pathophysiology. 2026 Jan 20;33(1):9. doi: 10.3390/pathophysiology33010009 (PMC12921874; doi:10.3390/pathophysiology33010009)
Supplement: Supplementary file 1 [file pathophysiology-33-00009-s001.zip › Supplementary Table 1.pdf]

Supplementary Table 1. The 170 pathogenic/likely pathogenic germline mutations in 64 male breast cancer cases.

ID	Gene	Systematic nomenclature	HGVS protein change	Annotation	Clinical significance
ly6862	FBN1	c.3463G>T	p.Asp1155Tyr	missense_variant&splice_region_variant	Likely_pathogenic
ly6860	TCHH	c.991C>G	p.Gln331Glu	missense_variant	Pathogenic
ly6860	LRP6	c.3178C>A		protein_protein_contact	Pathogenic
ly6861	TRPS1	c.3425G>T	p.Ser1142Ile	missense_variant	Pathogenic
ly6861	CCDC103	c.383delG	p.Gly128fs	frameshift_variant	Pathogenic
ly6861	DUOX2	c.3329G>A	p.Arg1110Gln	missense_variant	Pathogenic
ly6861	MYBPC1	c.667G>T	p.Glu223*	stop_gained	Pathogenic
ly6862	PMPCA	c.287C>A	p.Ser96*	stop_gained	Pathogenic
ly6862	RECQL4	c.1015delC	p.Leu339fs	frameshift_variant	Pathogenic
ly6862	GJB2	c.95G>A	p.Arg32His	missense_variant	Pathogenic
ly6863	OTOF	c.4275G>A	p.Trp1425*	stop_gained	Pathogenic
ly6859	FAH	c.782C>T	p.Pro261Leu	missense_variant	Pathogenic/Likely_pathogenic
ly6865	PROM1	c.1697delA	p.Asn566fs	frameshift_variant	Likely_pathogenic
ly6823	MRPS22	c.502C>T	p.Arg168Trp	missense_variant&splice_region_variant	Likely_pathogenic
ly6824	MEGF10	c.2362+2dupT		splice_donor_variant&splice_region_variant&intron_variant	Likely_pathogenic
ly6864	MRE11A	c.1906C>T	p.Arg636*	stop_gained	Pathogenic
ly6865	GJB1	c.547C>A	p.Arg183Ser	missense_variant	Pathogenic
ly6865	ANO10	c.132delA	p.Asp45fs	frameshift_variant	Pathogenic
ly6865	ZNF644	c.1759A>G	p.Ile587Val	missense_variant	Pathogenic

ly6865	ABCC6	c.3412C>T	p.Arg1138Trp	missense_variant	Pathogenic
ly6865	ERCC5	c.2751dupA	p.Leu918fs	frameshift_variant	Pathogenic
ly6866	POR	c.1479delC	p.Ile494fs	frameshift_variant	Pathogenic
ly6866	CPS1	c.505G>A	p.Gly169Arg	missense_variant	Pathogenic
ly6866	TSEN54	c.823delG	p.Val275fs	frameshift_variant	Pathogenic
ly6823	PITX1	c.388G>T	p.Glu130*	stop_gained	Pathogenic
ly6824	DH5	c.5563dupA	p.Ile1855fs	frameshift_variant	Pathogenic
ly6824	ANTXR2	c.1073dupC	p.Ala359fs	frameshift_variant	Pathogenic
ly6824	SLC17A9	c.25C>T	p.Arg9Cys	missense_variant	Pathogenic
ly6824	LRP6	c.1418G>A	p.Arg473Gln	missense_variant	Pathogenic
ly6864	MYBPC3	c.1624+4A>C		splice_donor_variant&splice_region_variant&intron_variant	Pathogenic/Likely_pathogenic
ly6824	RET	c.1853G>T	p.Cys618Phe	missense_variant	Pathogenic/Likely_pathogenic
ly6825	PEX1	c.1108delA	p.Ile370fs	frameshift_variant	Likely_pathogenic
ly6829	SACS	c.3328delA	p.Ile1110fs	frameshift_variant	Likely_pathogenic
ly6830	GBA	c.1603C>T	p.Arg535Cys	missense_variant	Likely_pathogenic
ly6825	SEC63	c.1605dupA	p.Pro536fs	frameshift_variant	Pathogenic
ly6828	STRA6	c.386C>T	p.Pro129Leu	missense_variant&splice_region_variant	Pathogenic
ly6829	GBA	c.586A>C	p.Lys196Gln	missense_variant&splice_region_variant	Pathogenic
ly6830	ATP2A1	c.2366C>T	p.Pro789Leu	missense_variant	Pathogenic
ly6832	LIPH	c.742C>A	p.His248Asn	missense_variant	Pathogenic
ly6828	AGXT	c.25_26insC		sequence_feature	Pathogenic
ly6825	DMD	c.4000G>T	p.Gly1334*	stop_gained	Pathogenic/Likely_pathogenic
ly6830	MUTYH	c.857G>A	p.Gly286Glu	missense_variant	Pathogenic/Likely_pathogenic
ly6832	CYP27A1	c.1214G>A	p.Arg405Gln	missense_variant	Pathogenic/Likely_pathogenic

ly6874	CDKN2A	c.338C>A	p.Pro113Gln	missense_variant	Likely_pathogenic
ly6874	LRPPRC	c.3147dupA	p.Gly1050fs	frameshift_variant&splice_region_variant	Pathogenic
ly6874	ZMPSTE24	c.1085dupT	p.Leu362fs	frameshift_variant	Pathogenic
ly6875	GALT	c.490C>A	p.Gln164Lys	missense_variant	Pathogenic
ly6875	ADAMTS13	c.4142C>A	p.Ser1381*	stop_gained	Pathogenic
ly6875	TGFB1	c.664C>A	p.His222Asn	missense_variant	Pathogenic
ly6876	ELN	c.1097-1G>T		splice_acceptor_variant&intron_variant	Pathogenic
ly6877	GRHPR	c.102G>T	p.Trp34Cys	missense_variant	Pathogenic
ly6878	COL7A1	c.4871delC	p.Pro1624fs	frameshift_variant	Pathogenic
ly6874	BCL10	c.136delA	p.Ile46fs	frameshift_variant	Pathogenic
ly6876	RAD50	c.2165delA	p.Lys722fs	frameshift_variant	Pathogenic
ly6874	SLC34A1	c.458G>T	p.Gly153Val	missense_variant	Pathogenic
ly6876	CPT2	c.452G>T	p.Arg151Leu	missense_variant	Pathogenic/Likely_pathogenic
ly6878	FBN1	c.7806G>T	p.Trp2602Cys	missense_variant	Pathogenic/Likely_pathogenic
ly6848	PCDHB4	c.915dupA	p.Leu306fs	frameshift_variant	Likely_pathogenic
ly6849	USH2A	c.12739G>T	p.Gly4247Trp	missense_variant	Likely_pathogenic
ly6849	MEN1	c.1366-1G>T		splice_acceptor_variant&intron_variant	Likely_pathogenic
ly6853	A10	c.339G>T	p.Lys113Asn	missense_variant&splice_region_variant	Likely_pathogenic
ly6853	RAD50	c.1245+2C>A		splice_donor_variant&intron_variant	Likely_pathogenic
ly6853	SLC22A5	c.202C>A	p.Pro68Thr	missense_variant	Likely_pathogenic
ly6856	AMPD2	c.1133G>A	p.Arg378Gln	missense_variant	Likely_pathogenic
ly6851	TNNI3	c.575G>A	p.Arg192His	missense_variant	Likely_pathogenic
ly6848	F8	c.4379dupA	p.Asn1460fs	frameshift_variant	Pathogenic
ly6849	IKBKG	c.1371delC	p.Glu458fs	frameshift_variant	Pathogenic

ly6849	KCNH2	c.2416G>T	p.Gly806Trp	missense_variant	Pathogenic
ly6849	CYP21A2	c.955C>T	p.Gln319*	stop_gained	Pathogenic
ly6849	MUTYH	c.799C>T	p.Gln267*	stop_gained	Pathogenic
ly6849	PLOD1	c.1792-3C>A		splice_acceptor_variant&splice_region_variant&intron_variant	Pathogenic
ly6849	ATP1A3	c.2600G>A	p.Gly867Asp	missense_variant	Pathogenic
ly6850	SMARCB1	c.143C>A	p.Pro48His	missense_variant	Pathogenic
ly6850	FUCA1	c.244C>A	p.Gln82Lys	missense_variant	Pathogenic
ly6851	FSHB	c.282C>G	p.Tyr94*	stop_gained	Pathogenic
ly6852	FBP1	c.704delC	p.Pro235fs	frameshift_variant&splice_region_variant	Pathogenic
ly6853	ARSA	c.302delG	p.Gly101fs	frameshift_variant	Pathogenic
ly6853	TMPRSS6	c.1786delG	p.Ala596fs	frameshift_variant	Pathogenic
ly6853	PEX12	c.538C>G	p.Arg180Gly	missense_variant	Pathogenic
ly6854	NTHL1	c.268C>G	p.Gln90Glu	missense_variant	Pathogenic
ly6854	LTBP3	c.2216delG	p.Gly739fs	frameshift_variant	Pathogenic
ly6855	SCN8A	c.1588C>T	p.Arg530Trp	missense_variant	Pathogenic
ly6856	DDX3X	c.1126C>A		protein_protein_contact	Pathogenic
ly6856	COL3A1	c.1231G>T	p.Gly411*	stop_gained	Pathogenic
ly6856	ADAMTSL4	c.2339delG	p.Gly780fs	frameshift_variant	Pathogenic
ly6856	MFRP	c.498dupC	p.Asn167fs	frameshift_variant	Pathogenic
ly6849	SLC26A4	c.2168A>G	p.His723Arg	missense_variant	Pathogenic/Likely_pathogenic
ly6853	GJB2	c.230G>T	p.Trp77Leu	missense_variant	Pathogenic/Likely_pathogenic
ly6857	KANSL1	c.3125delT	p.Leu1042fs	frameshift_variant	Likely_pathogenic
ly6858	SDHB	c.237delT	p.Lys80fs	frameshift_variant	Likely_pathogenic
ly6881	MLYCD	c.1295G>A	p.Arg432His	missense_variant	Likely_pathogenic

ly6882	RARS2	c.1055delA	p.Lys352fs	frameshift_variant	Likely_pathogenic
ly6885	NIPBL	c.4561-2A>G		splice_acceptor_variant&intron_variant	Likely_pathogenic
ly6885	FAH	c.960+1G>T		splice_donor_variant&intron_variant	Likely_pathogenic
ly6886	SCN2A	c.2695G>T	p.Gly899Cys	missense_variant	Likely_pathogenic
ly6886	VWF	c.7390C>A	p.Arg2464Ser	missense_variant	Likely_pathogenic
ly6886	DNM1L	c.475G>A	p.Asp159Asn	missense_variant	Likely_pathogenic
ly6886	CDH23	c.7936G>C	p.Asp2646His	missense_variant	Likely_pathogenic
ly6887	XRCC2	c.350dupT	p.Leu117fs	frameshift_variant	Likely_pathogenic
ly6857	AMPD3	c.1744C>T	p.Arg582Cys	missense_variant	Likely_pathogenic,_Affects
ly6857	MECP2	c.279delC	p.Lys94fs	frameshift_variant	Pathogenic
ly6857	RSPH1	c.727+5G>A		splice_donor_variant&splice_region_variant&intron_variant	Pathogenic
ly6858	LRP5	c.1828G>T	p.Gly610Trp	missense_variant	Pathogenic
ly6879	MPZ	c.679C>T	p.Pro227Ser	missense_variant	Pathogenic
ly6880	AHI1	c.910dupA	p.Thr304fs	frameshift_variant	Pathogenic
ly6880	SMPD1	c.564dupC	p.Lys189fs	frameshift_variant	Pathogenic
ly6883	SLC22A5	c.1472C>G	p.Ser491Cys	missense_variant	Pathogenic
ly6884	DKC1	c.1226C>T	p.Pro409Leu	missense_variant	Pathogenic
ly6884	AMELX	c.17delT	p.Leu6fs	frameshift_variant	Pathogenic
ly6885	DMD	c.5697delA	p.Lys1899fs	frameshift_variant	Pathogenic
ly6885	TUBB1	c.952C>T	p.Arg318Trp	missense_variant	Pathogenic
ly6886	FGD1	c.1223G>T	p.Arg408Leu	missense_variant	Pathogenic
ly6886	RS1	c.608C>A	p.Pro203Gln	missense_variant	Pathogenic
ly6886	TAZ	c.589G>T	p.Gly197Trp	missense_variant	Pathogenic
ly6886	SLC26A3	c.1386G>T	p.Trp462Cys	missense_variant	Pathogenic

ly6886	ELANE	c.598-1G>T		splice_acceptor_variant&intron_variant	Pathogenic
ly6886	CTSD	c.1196G>A	p.Arg399His	missense_variant	Pathogenic
ly6887	F8	c.4379delA	p.Asn1460fs	frameshift_variant	Pathogenic
ly6887	SUN5	c.381delA	p.Val128fs	frameshift_variant	Pathogenic
ly6887	PCSK9	c.1426C>G		protein_protein_contact	Pathogenic
ly6887	AGT	c.1290delT	p.Phe430fs	frameshift_variant	Pathogenic
ly6888	MMADHC	c.134C>A	p.Ala45Asp	missense_variant	Pathogenic
ly6888	B4GALNT1	c.263delG	p.Gly88fs	frameshift_variant	Pathogenic
ly6888	TH	c.698G>A	p.Arg233His	missense_variant	Pathogenic
ly6883	LRP2	c.13139delC	p.Pro4380fs	frameshift_variant	Pathogenic
ly6881	HPS1	c.972delC	p.Met325fs	frameshift_variant	Pathogenic
ly6884	ABCC6	c.1990C>T	p.Pro664Ser	missense_variant	Pathogenic
ly6886	BRCA2	c.5073delA	p.Lys1691fs	frameshift_variant	Pathogenic
ly6888	FLCN	c.1285delC	p.His429fs	frameshift_variant	Pathogenic
ly6887	MSH6	c.3261delC	p.Phe1088fs	frameshift_variant	Pathogenic
ly6886	DMD	c.5530C>T	p.Arg1844*	stop_gained	Pathogenic
ly6858	ACADS	c.1031A>G	p.Glu344Gly	missense_variant&splice_region_variant	Pathogenic/Likely_pathogenic
ly6883	PPT1	c.169delA		protein_protein_contact	Pathogenic/Likely_pathogenic
ly6885	REEP1	c.533delC	p.Pro178fs	frameshift_variant	Pathogenic/Likely_pathogenic
ly6887	PROKR2	c.533G>C	p.Trp178Ser	missense_variant	Pathogenic/Likely_pathogenic
ly6764	SLC26A4	c.365delT	p.Phe122fs	frameshift_variant	Likely_pathogenic
ly6818	OCA2	c.1211C>T	p.Thr404Met	missense_variant	Likely_pathogenic
ly6821	GFM2	c.569G>A	p.Arg190Gln	missense_variant	Likely_pathogenic
ly6871	WDR62	c.1480G>A	p.Gly494Arg	missense_variant	Likely_pathogenic

ly6872	TMPRSS3	c.598G>A	p.Val200Met	missense_variant	Likely_pathogenic
ly6873	SCN1A	c.1660C>A	p.Gln554Lys	missense_variant&splice_region_variant	Likely_pathogenic
ly6820	ARSA	c.418delC	p.His140fs	frameshift_variant	Likely_pathogenic
ly6870	ARSA	c.1492delC	p.Arg498fs	frameshift_variant	Likely_pathogenic
ly6870	TP53	c.817dupC		protein_protein_contact	Likely_pathogenic
ly6761	OSMR	c.2090A>C	p.Lys697Thr	missense_variant	Pathogenic
ly6761	ABCG5	c.1166G>A	p.Arg389His	missense_variant	Pathogenic
ly6764	TGFBI	c.1501C>A	p.Pro501Thr	missense_variant	Pathogenic
ly6764	ATP7B	c.4114C>T	p.Gln1372*	stop_gained	Pathogenic
ly6817	CYP4V2	c.1091-2A>G		splice_acceptor_variant&intron_variant	Pathogenic
ly6817	DBT	c.75_76delAT	p.Cys26fs	frameshift_variant	Pathogenic
ly6817	SLC24A5	c.1361dupT	p.Leu454fs	frameshift_variant	Pathogenic
ly6817	ATP7B	c.2975C>T	p.Pro992Leu	missense_variant	Pathogenic
ly6820	ABCC6	c.196dupT	p.Ser66fs	frameshift_variant	Pathogenic
ly6821	FRAS1	c.2722+1G>T		splice_donor_variant&intron_variant	Pathogenic
ly6821	BRCA2	c.3387G>C	p.Gln1129His	missense_variant	Pathogenic
ly6870	TEX11	c.511A>G	p.Met171Val	missense_variant	Pathogenic
ly6870	FBXL4	c.292C>T	p.Arg98*	stop_gained	Pathogenic
ly6870	KLHL40	c.1582G>A		protein_protein_contact	Pathogenic
ly6871	FMO3	c.591_592delTG	p.Cys197fs	frameshift_variant	Pathogenic
ly6872	SLC26A8	c.2860C>T	p.Arg954Cys	missense_variant	Pathogenic
ly6872	OTOF	c.1621G>T	p.Gly541Cys	missense_variant	Pathogenic
ly6873	SLC12A3	c.179C>T	p.Thr60Met	missense_variant	Pathogenic
ly6873	HCN4	c.1438G>T	p.Gly480Cys	missense_variant	Pathogenic

ly6893	GLB1	c.1588C>T	p.Arg530Cys	missense_variant	Pathogenic
ly6893	PLCE1	c.3346C>G	p.Arg1116Gly	missense_variant	Pathogenic
ly6762	RAD50	c.2165dupA	p.Glu723fs	frameshift_variant	Pathogenic
ly6891	GNPTAB	c.2693dupA	p.Tyr899fs	frameshift_variant	Pathogenic
ly6893	CTC-435M10.3	c.219delC	p.Arg74fs	frameshift_variant	Pathogenic
ly6818	USH2A	c.2802T>G	p.Cys934Trp	missense_variant	Pathogenic/Likely_pathogenic
ly6873	SGCA	c.850C>A	p.Arg284Ser	missense_variant	Pathogenic/Likely_pathogenic
