# Supplementary material for: Novel Insights into the Enigmatic Genetics of Male Breast Cancer in China
Source: Pathophysiology. 2026 Jan 20;33(1):9. doi: 10.3390/pathophysiology33010009 (PMC12921874; doi:10.3390/pathophysiology33010009)
Supplement: Supplementary file 1 [file pathophysiology-33-00009-s001.zip › Supplementary Table 2.pdf]

Supplementary Table 2. The 413 VUS germline mutations in 64 male breast cancer cases.

ID	Gene	Systematic nomenclature	HGVS protein change	Annotation	Clinical significance
ly6760	COL7A1	c.3182C>T	p.Ala1061Val	missense_variant	Uncertain_significance
ly6760	PANK2	c.1133A>G	p.Asp378Gly	missense_variant	Uncertain_significance
ly6760	RAB3GAP2	c.1205C>G	p.Thr402Arg	missense_variant	Uncertain_significance
ly6760	CASQ2	c.34T>A	p.Tyr12Asn	missense_variant	Uncertain_significance
ly6760	LOXHD1	c.2227A>G	p.Thr743Ala	missense_variant	Uncertain_significance
ly6760	ZIC2	c.1389_1406delGGCTGCGGCGGCGGCGGC	p.Ala464_Ala469del	disruptive_inframe_deletion	Uncertain_significance
ly6760	CACNA1C	c.5329C>T	p.Arg1777Cys	missense_variant	Uncertain_significance
ly6760	MYH7	c.5704G>C	p.Glu1902Gln	missense_variant	Uncertain_significance
ly6761	CFTR	c.2597G>A	p.Cys866Tyr	missense_variant	Uncertain_significance
ly6761	SLC30A10	c.676A>G	p.Met226Val	missense_variant	Uncertain_significance
ly6761	SDHD	c.328G>A	p.Val110Ile	missense_variant	Uncertain_significance
ly6762	PRSS12	c.1357G>A	p.Val453Ile	missense_variant	Uncertain_significance
ly6762	ABCA4	c.5714+4C>T		splice_donor_variant&splice_region_variant&intron_variant	Uncertain_significance
ly6762	CDH1	c.2194C>T	p.Arg732Trp	missense_variant	Uncertain_significance
ly6763	GRIK2	c.1360T>C	p.Tyr454His	missense_variant	Uncertain_significance
ly6763	SCN5A	c.3556G>A	p.Ala1186Thr	missense_variant	Uncertain_significance
ly6763	FAM83C	c.863G>A	p.Arg288His	missense_variant	Uncertain_significance

ly6763	SPTA1	c.3697G>A	p.Val1233Ile	missense_variant	Uncertain_significance
ly6763	TSC2	c.3610+4C>T		splice_donor_variant&splice_region_variant&intron_variant	Uncertain_significance
ly6763	L2HGDH	c.1174A>G	p.Ile392Val	missense_variant	Uncertain_significance
ly6763	MYBPC3	c.104G>A	p.Arg35Gln	missense_variant	Uncertain_significance
ly6764	TG	c.1532A>G	p.Asn511Ser	missense_variant	Uncertain_significance
ly6764	ADAMTS2	c.2914C>A	p.Arg972Ser	missense_variant	Uncertain_significance
ly6764	DOK7	c.1313C>G	p.Thr438Arg	missense_variant	Uncertain_significance
ly6764	BSND	c.16A>G	p.Thr6Ala	missense_variant	Uncertain_significance
ly6764	KL	c.911A>G	p.Asn304Ser	missense_variant	Uncertain_significance
ly6817	SLC26A2	c.1512G>A	p.Met504Ile	missense_variant	Uncertain_significance
ly6817	DRC1	c.1622A>G	p.Asp541Gly	missense_variant	Uncertain_significance
ly6817	TTN	c.88106G>T	p.Gly29369Val	missense_variant	Uncertain_significance
ly6817	MYO15A	c.5964+3G>A		splice_donor_variant&splice_region_variant&intron_variant	Uncertain_significance
ly6817	ZNF469	c.1781C>T	p.Pro594Leu	missense_variant	Uncertain_significance
ly6818	QARS	c.601C>T	p.Arg201Trp	missense_variant	Uncertain_significance
ly6818	UROC1	c.40C>T	p.Arg14Trp	missense_variant	Uncertain_significance
ly6818	KIF21B	c.2224G>A	p.Glu742Lys	missense_variant	Uncertain_significance
ly6818	POMT2	c.871C>G	p.Leu291Val	missense_variant	Uncertain_significance
ly6818	NIN	c.421G>T	p.Gly141Cys	missense_variant	Uncertain_significance
ly6819	EVC	c.1852G>A	p.Gly618Ser	missense_variant	Uncertain_significance

ly6819	FGA	c.923G>A	p.Arg308Gln	missense_variant	Uncertain_significance
ly6819	ARHGAP31	c.3461C>T	p.Pro1154Leu	missense_variant	Uncertain_significance
ly6819	DYSF	c.4859G>A	p.Arg1620His	missense_variant	Uncertain_significance
ly6819	TTN	c.107080C>G	p.Leu35694Val	missense_variant	Uncertain_significance
ly6820	SURF1	c.352A>T	p.Arg118Trp	missense_variant	Uncertain_significance
ly6820	MCC	c.800G>A	p.Arg267His	missense_variant	Uncertain_significance
ly6820	SLC26A1	c.433G>A	p.Gly145Ser	missense_variant	Uncertain_significance
ly6820	DPYD	c.1774C>T	p.Arg592Trp	missense_variant	Uncertain_significance
ly6820	ALX4	c.728C>T	p.Ala243Val	missense_variant	Uncertain_significance
ly6820	CBL	c.1459A>G	p.Met487Val	missense_variant	Uncertain_significance
ly6821	DFNB31	c.1892C>T	p.Ala631Val	missense_variant	Uncertain_significance
ly6821	CNGB3	c.1531G>A	p.Ala511Thr	missense_variant	Uncertain_significance
ly6821	UPB1	c.889G>A	p.Glu297Lys	missense_variant	Uncertain_significance
ly6821	DNMT3B	c.1804G>A	p.Val602Ile	missense_variant	Uncertain_significance
ly6821	SPTA1	c.480C>G	p.Phe160Leu	missense_variant	Uncertain_significance
ly6821	CEP152	c.4175T>C	p.Ile1392Thr	missense_variant	Uncertain_significance
ly6821	PRKAG2	c.593delC	p.Pro198fs	frameshift_variant	Uncertain_significance
ly6821	MYH7	c.4985G>A	p.Arg1662His	missense_variant	Uncertain_significance
ly6822	APC	c.794G>A	p.Gly265Glu	missense_variant	Uncertain_significance
ly6822	WFS1	c.535G>A	p.Ala179Thr	missense_variant	Uncertain_significance
ly6822	NPHP4	c.2849G>A	p.Arg950Gln	missense_variant	Uncertain_significance
ly6822	MAP2K2	c.281C>T	p.Ser94Leu	missense_variant	Uncertain_significance
ly6822	TNFRSF13B	c.592C>T	p.Arg198Cys	missense_variant	Uncertain_significance
ly6822	MYO7A	c.3503G>A	p.Arg1168Gln	missense_variant&splice_regi	Uncertain_significance

				on_variant	
ly6822	KCNN3	c.239_241dupAGC	p.Gln80dup	inframe_insertion	Uncertain_significance
ly6823	RP1L1	c.4027_4029delGAA	p.Glu1343del	inframe_deletion	Uncertain_significance
ly6823	SLC12A6	c.2003G>T	p.Arg668Leu	missense_variant	Uncertain_significance
ly6824	FLNA	c.7612C>A	p.Leu2538Met	missense_variant	Uncertain_significance
ly6824	DNAJB6	c.836_841dupAGGAGG	p.Glu279_Glu280dup	disruptive_inframe_insertion	Uncertain_significance
ly6824	APOB	c.11443G>A	p.Val3815Met	missense_variant	Uncertain_significance
ly6824	CANT1	c.556G>A	p.Val186Ile	missense_variant	Uncertain_significance
ly6824	POLE	c.4337_4338delTG	p.Val1446fs	frameshift_variant	Uncertain_significance
ly6825	PRKAG2	c.206C>G	p.Pro69Arg	missense_variant	Uncertain_significance
ly6825	SYNE1	c.16025C>T	p.Thr5342Met	missense_variant	Uncertain_significance
ly6825	FBN2	c.2717G>A	p.Arg906His	missense_variant	Uncertain_significance
ly6825	TTN	c.24454G>A	p.Val8152Ile	missense_variant	Uncertain_significance
ly6825	ADSL	c.967C>A	p.Arg323Ser	missense_variant	Uncertain_significance
ly6825	OTOA	c.1214C>T	p.Ser405Leu	missense_variant	Uncertain_significance
ly6825	SPTB	c.5810C>G	p.Ser1937Cys	missense_variant	Uncertain_significance
ly6825	CNTNAP2	c.2570C>A	p.Ser857Tyr	missense_variant	Uncertain_significance
ly6826	NBEAL2	c.4367G>A	p.Arg1456His	missense_variant	Uncertain_significance
ly6826	TTN	c.70492G>A	p.Gly23498Ser	missense_variant	Uncertain_significance
ly6826	SLC5A7	c.1306G>A	p.Val436Met	missense_variant	Uncertain_significance
ly6826	PLCB4	c.539delA	p.Lys180fs	frameshift_variant	Uncertain_significance
ly6826	ACTN2	c.1984C>T	p.Arg662Trp	missense_variant	Uncertain_significance
ly6826	ITGB4	c.1805A>T	p.His602Leu	missense_variant	Uncertain_significance
ly6826	BRCA1	c.3356C>G	p.Thr1119Ser	missense_variant	Uncertain_significance

ly6827	FGG	c.274C>T	p.Leu92Phe	missense_variant	Uncertain_significance
ly6827	CERKL	c.1205A>C	p.Gln402Pro	missense_variant	Uncertain_significance
ly6827	SLC5A1	c.1556C>T	p.Thr519Met	missense_variant	Uncertain_significance
ly6827	CHEK2	c.1567G>A	p.Ala523Thr	missense_variant	Uncertain_significance
ly6827	SDHB	c.709C>T	p.Pro237Ser	missense_variant	Uncertain_significance
ly6827	CHRNB1	c.565G>A	p.Gly189Arg	missense_variant	Uncertain_significance
ly6827	PALB2	c.2360C>T	p.Thr787Ile	missense_variant	Uncertain_significance
ly6827	ERCC5	c.2890C>T	p.Arg964Trp	missense_variant	Uncertain_significance
ly6828	RECQL4	c.2747C>A	p.Pro916Gln	missense_variant	Uncertain_significance
ly6828	FANCM	c.1741C>T	p.Arg581Cys	missense_variant	Uncertain_significance
ly6828	CLIP1	c.4066G>A	p.Gly1356Arg	missense_variant	Uncertain_significance
ly6829	PGAP3	c.827C>T	p.Pro276Leu	missense_variant	Uncertain_significance
ly6829	CTSD	c.154G>A	p.Val52Ile	missense_variant	Uncertain_significance
ly6829	RBM20	c.1784A>G	p.Lys595Arg	missense_variant	Uncertain_significance
ly6830	RAD50	c.1211A>G	p.Gln404Arg	missense_variant	Uncertain_significance
ly6830	FBN2	c.3394G>A	p.Val1132Ile	missense_variant	Uncertain_significance
ly6830	TRIOBP	c.5266C>T	p.Arg1756Trp	missense_variant	Uncertain_significance
ly6830	POLD1	c.2542G>T	p.Asp848Tyr	missense_variant	Uncertain_significance
ly6830	BRIP1	c.1357G>A	p.Ala453Thr	missense_variant	Uncertain_significance
ly6830	BRCA2	c.3403T>C	p.Tyr1135His	missense_variant	Uncertain_significance
ly6832	INPP5E	c.875G>A	p.Arg292His	missense_variant	Uncertain_significance
ly6832	AARS2	c.2701C>T	p.Arg901Trp	missense_variant	Uncertain_significance
ly6832	SMARCAL1	c.1413C>G	p.Ile471Met	missense_variant	Uncertain_significance
ly6832	ACTN2	c.1552C>T		protein_protein_contact	Uncertain_significance

ly6832	HPD	c.5C>T	p.Thr2Met	missense_variant&splice_region_variant	Uncertain_significance
ly6832	MSH6	c.1501C>T	p.His501Tyr	missense_variant	Uncertain_significance
ly6847	MSH6	c.2875C>T	p.Arg959Cys	missense_variant	Uncertain_significance
ly6847	COL6A3	c.9130G>A	p.Val3044Ile	missense_variant	Uncertain_significance
ly6847	SMARCAL1	c.2765T>C	p.Met922Thr	missense_variant	Uncertain_significance
ly6847	MMADHC	c.515A>C	p.Lys172Thr	missense_variant	Uncertain_significance
ly6847	EP300	c.2242-5_2242-4delTT		splice_acceptor_variant&splice_region_variant&intron_variant	Uncertain_significance
ly6847	PEX14	c.993T>G	p.Asp331Glu	missense_variant	Uncertain_significance
ly6847	POLD1	c.946G>A	p.Asp316Asn	missense_variant	Uncertain_significance
ly6847	TGM5	c.167A>T	p.Asn56Ile	missense_variant	Uncertain_significance
ly6847	MYO7A	c.4757A>G	p.Asn1586Ser	missense_variant	Uncertain_significance
ly6847	OTOG	c.952G>A	p.Ala318Thr	missense_variant	Uncertain_significance
ly6848	DDX26B	c.359delT	p.Leu120fs	frameshift_variant	Uncertain_significance
ly6848	TJP2	c.804_809dupTGACCG	p.Arg270_Ser271insAspArg	disruptive_inframe_insertion	Uncertain_significance
ly6848	SLC27A5	c.1160G>T	p.Arg387Leu	missense_variant	Uncertain_significance
ly6849	MED12	c.3785G>A	p.Arg1262Lys	missense_variant	Uncertain_significance
ly6849	TRAPPC9	c.3215G>A	p.Arg1072Gln	missense_variant	Uncertain_significance
ly6849	FBXL4	c.559C>A	p.Gln187Lys	missense_variant	Uncertain_significance
ly6849	IYD	c.874C>A	p.Arg292Ser	missense_variant	Uncertain_significance
ly6849	NBEAL2	c.1618delC	p.Arg540fs	frameshift_variant	Uncertain_significance
ly6849	SUCLG1	c.242A>G		sequence_feature	Uncertain_significance

ly6849	COL6A3	c.8634_8651delTACGACGAAGCCGGTGAC	p.Thr2879_Thr2884del	disruptive_inframe_deletion	Uncertain_significance
ly6849	NDUFAF5	c.264-3dupT		splice_acceptor_variant&intron_variant	Uncertain_significance
ly6849	ZNF469	c.248C>A	p.Pro83Gln	missense_variant	Uncertain_significance
ly6849	STK11	c.125G>T	p.Arg42Leu	missense_variant	Uncertain_significance
ly6850	TTI2	c.695C>G	p.Thr232Ser	missense_variant	Uncertain_significance
ly6850	SYNE1	c.23C>T	p.Ser8Phe	missense_variant	Uncertain_significance
ly6850	RAD50	c.1592A>G	p.His531Arg	missense_variant	Uncertain_significance
ly6850	F11	c.219-4dupA		splice_acceptor_variant&splice_region_variant&intron_variant	Uncertain_significance
ly6850	SCN10A	c.3704C>T	p.Ala1235Val	missense_variant	Uncertain_significance
ly6850	COL6A1	c.356C>T	p.Ala119Val	missense_variant	Uncertain_significance
ly6850	SLC30A10	c.1249C>A	p.Pro417Thr	missense_variant	Uncertain_significance
ly6850	CHIT1	c.1352C>A	p.Pro451Gln	missense_variant	Uncertain_significance
ly6850	HMCN1	c.7079G>A	p.Arg2360His	missense_variant	Uncertain_significance
ly6850	AGL	c.4331A>G	p.Asn1444Ser	missense_variant	Uncertain_significance
ly6850	DLG4	c.735G>T	p.Lys245Asn	missense_variant	Uncertain_significance
ly6850	POLE	c.3019G>T	p.Ala1007Ser	missense_variant	Uncertain_significance
ly6850	RBM20	c.2359G>A	p.Glu787Lys	missense_variant	Uncertain_significance
ly6850	NEB	c.20467-4_20467-3insTA		splice_acceptor_variant&splice_region_variant&intron_variant	Uncertain_significance
ly6851	ADAMTS2	c.2015G>T	p.Arg672Leu	missense_variant	Uncertain_significance

ly6851	TTN	c.75997G>T	p.Gly25333Cys	missense_variant	Uncertain_significance
ly6851	CNGB1	c.1631C>T	p.Pro544Leu	missense_variant	Uncertain_significance
ly6851	ZC3H14	c.1851_1868+13delCCCCATCTCACCCTGCAAGTGAGTACCATCC		sequence_feature	Uncertain_significance
ly6852	MYO5B	c.2644C>T	p.Arg882Trp	missense_variant	Uncertain_significance
ly6852	BRIP1	c.1688A>G	p.Asp563Gly	missense_variant	Uncertain_significance
ly6852	PTPN11	c.1124A>G	p.Tyr375Cys	missense_variant	Uncertain_significance
ly6852	MRPS16	c.59G>A	p.Arg20His	missense_variant	Uncertain_significance
ly6853	FLNA	c.2123G>A	p.Arg708Gln	missense_variant	Uncertain_significance
ly6853	CDK5RAP2	c.3002C>T		sequence_feature	Uncertain_significance
ly6853	DSP	c.1942G>A	p.Asp648Asn	missense_variant	Uncertain_significance
ly6853	APC	c.1408+5G>A		splice_donor_variant&splice_region_variant&intron_variant	Uncertain_significance
ly6853	EVC2	c.887G>T	p.Gly296Val	missense_variant	Uncertain_significance
ly6853	ALK	c.386G>T	p.Gly129Val	missense_variant	Uncertain_significance
ly6853	KIF1B	c.4683C>A	p.Ser1561Arg	missense_variant	Uncertain_significance
ly6853	MYO1A	c.829G>T	p.Val277Leu	missense_variant	Uncertain_significance
ly6853	BRCA1	c.5348G>T	p.Arg1783Met	missense_variant	Uncertain_significance
ly6854	DYSF	c.4037C>T	p.Ala1346Val	missense_variant	Uncertain_significance
ly6854	TTN	c.74527A>G	p.Asn24843Asp	missense_variant	Uncertain_significance
ly6854	FOXN1	c.1216G>A	p.Gly406Ser	missense_variant	Uncertain_significance
ly6854	CDH23	c.9919G>A	p.Glu3307Lys	missense_variant	Uncertain_significance
ly6854	PCDH15	c.4320_4322delGCC	p.Pro1441del	disruptive_inframe_deletion	Uncertain_significance

ly6854	MSH2	c.2099C>A	p.Ala700Glu	missense_variant	Uncertain_significance
ly6855	GRHPR	c.682A>G	p.Lys228Glu	missense_variant	Uncertain_significance
ly6855	ADAMTS13	c.540-3G>A		splice_acceptor_variant&splice_region_variant&intron_variant	Uncertain_significance
ly6855	C5orf42	c.608A>G	p.Tyr203Cys	missense_variant	Uncertain_significance
ly6855	HMCN1	c.4828G>A	p.Val1610Ile	missense_variant	Uncertain_significance
ly6856	TYRP1	c.785C>T	p.Thr262Met	missense_variant	Uncertain_significance
ly6856	VCAN	c.8053G>A	p.Val2685Ile	missense_variant	Uncertain_significance
ly6856	LRBA	c.2516G>A	p.Arg839His	missense_variant	Uncertain_significance
ly6856	SCN5A	c.5216G>A	p.Arg1739Gln	missense_variant	Uncertain_significance
ly6856	MYLK	c.1213A>G	p.Met405Val	missense_variant	Uncertain_significance
ly6856	TTN	c.59236G>T	p.Gly19746Cys	missense_variant	Uncertain_significance
ly6856	PLEKHG5	c.1231C>T	p.Arg411Trp	missense_variant	Uncertain_significance
ly6856	CACNA1H	c.3469C>A	p.Arg1157Ser	missense_variant	Uncertain_significance
ly6857	TPRN	c.1468G>A	p.Val490Met	missense_variant	Uncertain_significance
ly6857	SLC26A4	c.1983C>A	p.Asp661Glu	missense_variant	Uncertain_significance
ly6857	LAMA4	c.2912C>T	p.Ser971Phe	missense_variant	Uncertain_significance
ly6857	COL7A1	c.3232C>T	p.Arg1078Cys	missense_variant	Uncertain_significance
ly6857	RPGRIP1L	c.962G>A	p.Arg321His	missense_variant	Uncertain_significance
ly6857	CDH23	c.6926G>A	p.Arg2309Gln	missense_variant	Uncertain_significance
ly6858	TNC	c.2491G>A		protein_protein_contact	Uncertain_significance
ly6858	OBSL1	c.4441G>A	p.Val1481Met	missense_variant	Uncertain_significance
ly6858	MASP2	c.967A>C	p.Ser323Arg	missense_variant	Uncertain_significance

ly6858	TGM1	c.2221G>A	p.Val741Ile	missense_variant	Uncertain_significance
ly6858	NEBL	c.561G>C	p.Gln187His	missense_variant	Uncertain_significance
ly6859	PDE6B	c.485C>A	p.Ser162*	stop_gained	Uncertain_significance
ly6859	ELAC2	c.2372G>A	p.Arg791Gln	missense_variant	Uncertain_significance
ly6859	TUBB3	c.667C>T	p.Arg223Trp	missense_variant	Uncertain_significance
ly6859	FAS	c.444-5delT		splice_acceptor_variant&splice_region_variant&intron_variant	Uncertain_significance
ly6859	NEBL	c.1225G>T	p.Glu409*	stop_gained&splice_region_variant	Uncertain_significance
ly6859	ABCC2	c.4447T>G	p.Phe1483Val	missense_variant	Uncertain_significance
ly6860	MCPH1	c.445G>A	p.Val149Ile	missense_variant	Uncertain_significance
ly6860	LAMB1	c.2676G>C	p.Gln892His	missense_variant	Uncertain_significance
ly6860	CYP21A2	c.740delA	p.Glu247fs	frameshift_variant&splice_region_variant	Uncertain_significance
ly6860	TTN	c.57242T>C	p.Ile19081Thr	missense_variant	Uncertain_significance
ly6860	FTCD	c.430G>A	p.Gly144Arg	missense_variant	Uncertain_significance
ly6860	HMCN1	c.5669T>C	p.Leu1890Ser	missense_variant	Uncertain_significance
ly6860	PKD1	c.6395T>G	p.Phe2132Cys	missense_variant	Uncertain_significance
ly6860	TSC2	c.5266G>A	p.Glu1756Lys	missense_variant	Uncertain_significance
ly6860	POLG	c.3650C>T	p.Ala1217Val	missense_variant	Uncertain_significance
ly6860	DLAT	c.991C>G	p.Pro331Ala	missense_variant	Uncertain_significance
ly6861	CFTR	c.2042A>T	p.Glu681Val	missense_variant	Uncertain_significance
ly6861	ALDH7A1	c.1016A>G	p.His339Arg	missense_variant	Uncertain_significance
ly6861	MMAA	c.494A>G	p.Lys165Arg	missense_variant	Uncertain_significance

ly6861	CEACAM16	c.208G>A	p.Val70Met	missense_variant	Uncertain_significance
ly6861	WRAP53	c.1565delC	p.Ala522fs	frameshift_variant	Uncertain_significance
ly6861	POLG	c.3122G>T	p.Trp1041Leu	missense_variant	Uncertain_significance
ly6861	SLCO1B3	c.1863dupT	p.Gly622fs	frameshift_variant&splice_region_variant	Uncertain_significance
ly6862	RP1L1	c.3431C>T	p.Ser1144Phe	missense_variant	Uncertain_significance
ly6862	LAMA2	c.4993G>A	p.Gly1665Arg	missense_variant	Uncertain_significance
ly6862	TTC37	c.3015-5_3015-4delTT		splice_acceptor_variant&splice_region_variant&intron_variant	Uncertain_significance
ly6862	SCN5A	c.677C>T	p.Ala226Val	missense_variant	Uncertain_significance
ly6862	TTN	c.24358C>T	p.Pro8120Ser	missense_variant	Uncertain_significance
ly6862	TTN	c.45725G>A	p.Arg15242Lys	missense_variant	Uncertain_significance
ly6862	BCR	c.3316G>A	p.Asp1106Asn	missense_variant	Uncertain_significance
ly6862	PCNT	c.3220C>T	p.Arg1074Trp	missense_variant	Uncertain_significance
ly6862	RYR1	c.6016-5C>A		splice_acceptor_variant&splice_region_variant&intron_variant	Uncertain_significance
ly6862	RP11-156P1.2	c.206G>T	p.Arg69Leu	missense_variant&splice_region_variant	Uncertain_significance
ly6862	ABCC6	c.3739C>A	p.Pro1247Thr	missense_variant	Uncertain_significance
ly6863	ETFDH	c.142A>G	p.Ile48Val	missense_variant	Uncertain_significance
ly6863	ARFGEF2	c.3758-3delC		splice_acceptor_variant&splice_region_variant&intron_variant	Uncertain_significance

ly6863	USH2A	c.7000A>G	p.Asn2334Asp	missense_variant	Uncertain_significance
ly6863	POLD1	c.1594G>A	p.Ala532Thr	missense_variant	Uncertain_significance
ly6863	FANCA	c.2395C>T	p.Pro799Ser	missense_variant	Uncertain_significance
ly6864	PKHD1	c.11131G>A	p.Gly3711Arg	missense_variant	Uncertain_significance
ly6864	MYO15A	c.7502C>T	p.Thr2501Met	missense_variant	Uncertain_significance
ly6864	TK2	c.880C>T	p.Arg294Trp	missense_variant	Uncertain_significance
ly6864	GPC6	c.1601G>A	p.Arg534His	missense_variant	Uncertain_significance
ly6865	ZNF711	c.1055-3delT		splice_acceptor_variant&splice_region_variant&intron_variant	Uncertain_significance
ly6865	FANCC	c.1000C>T	p.Arg334Trp	missense_variant	Uncertain_significance
ly6865	MAN1B1	c.689T>C	p.Phe230Ser	missense_variant	Uncertain_significance
ly6865	RECQL4	c.2411G>A	p.Arg804Gln	missense_variant	Uncertain_significance
ly6865	EYS	c.1460-5dupT		splice_acceptor_variant&splice_region_variant&intron_variant	Uncertain_significance
ly6865	NDUFA2	c.191A>G		sequence_feature	Uncertain_significance
ly6865	PNKP	c.959C>T	p.Pro320Leu	missense_variant	Uncertain_significance
ly6865	CCDC40	c.2968G>A	p.Asp990Asn	missense_variant	Uncertain_significance
ly6865	DACH1	c.247_249dupGGC	p.Gly83dup	inframe_insertion	Uncertain_significance
ly6865	MET	c.3119G>A	p.Arg1040Gln	missense_variant	Uncertain_significance
ly6866	DNAI1	c.1020-5dupT		splice_acceptor_variant&splice_region_variant&intron_variant	Uncertain_significance
ly6866	PLEC	c.11212C>T	p.Arg3738Trp	missense_variant	Uncertain_significance

ly6866	DLD	c.865delA	p.Ile289fs	frameshift_variant	Uncertain_significance
ly6866	TFR2	c.1528G>A	p.Ala510Thr	missense_variant	Uncertain_significance
ly6866	DNAH8	c.4714G>A	p.Glu1572Lys	missense_variant	Uncertain_significance
ly6866	MCCC1	c.1894C>T	p.Pro632Ser	missense_variant	Uncertain_significance
ly6866	TTN	c.32194G>T	p.Glu10732*	stop_gained	Uncertain_significance
ly6866	MRE11A	c.1268A>C	p.Lys423Thr	missense_variant	Uncertain_significance
ly6870	ADAMTS2	c.724G>A	p.Ala242Thr	missense_variant	Uncertain_significance
ly6870	RAD51C	c.1096C>T	p.Arg366Trp	missense_variant	Uncertain_significance
ly6870	SACS	c.2643G>C	p.Glu881Asp	missense_variant	Uncertain_significance
ly6870	MYO7A	c.4450C>A	p.Leu1484Ile	missense_variant	Uncertain_significance
ly6870	SDHB	c.352G>A	p.Asp118Asn	missense_variant	Uncertain_significance
ly6871	TSC1	c.593A>G	p.Asn198Ser	missense_variant	Uncertain_significance
ly6871	GPR98	c.5576A>G	p.His1859Arg	missense_variant	Uncertain_significance
ly6871	HSPG2	c.6011G>A	p.Arg2004His	missense_variant	Uncertain_significance
ly6871	ERCC5	c.2995T>G	p.Leu999Val	missense_variant	Uncertain_significance
ly6871	NDUFS8	c.598G>A	p.Ala200Thr	missense_variant	Uncertain_significance
ly6871	ARMC4	c.2011G>T	p.Ala671Ser	missense_variant	Uncertain_significance
ly6872	KANK1	c.1652G>T	p.Cys551Phe	missense_variant	Uncertain_significance
ly6872	FRAS1	c.3058C>T	p.Arg1020Cys	missense_variant	Uncertain_significance
ly6872	TBX15	c.980G>A	p.Arg327His	missense_variant	Uncertain_significance
ly6872	WDR62	c.3559G>A	p.Val1187Met	missense_variant	Uncertain_significance
ly6872	ATP7B	c.676C>T	p.Arg226Trp	missense_variant	Uncertain_significance
ly6873	COL5A1	c.5293C>T	p.Arg1765Cys	missense_variant	Uncertain_significance
ly6873	GLI3	c.1346G>T	p.Arg449Leu	missense_variant	Uncertain_significance

ly6873	MYLK2	c.1741C>A	p.Arg581Ser	missense_variant	Uncertain_significance
ly6873	MYO7A	c.1945C>T	p.Arg649Trp	missense_variant	Uncertain_significance
ly6873	ATM	c.5624G>T	p.Arg1875Leu	missense_variant	Uncertain_significance
ly6873	RBP3	c.787G>T	p.Ala263Ser	missense_variant	Uncertain_significance
ly6873	SCN5A	c.4018G>A	p.Val1340Ile	missense_variant	Uncertain_significance
ly6874	SRPX2	c.257G>A	p.Arg86His	missense_variant	Uncertain_significance
ly6874	ATP7A	c.1030A>G	p.Arg344Gly	missense_variant	Uncertain_significance
ly6874	PAK3	c.101C>A	p.Pro34Gln	missense_variant	Uncertain_significance
ly6874	AP5Z1	c.1334C>T	p.Pro445Leu	missense_variant	Uncertain_significance
ly6874	MET	c.3253C>A	p.Gln1085Lys	missense_variant	Uncertain_significance
ly6874	EYS	c.1460-5delT		splice_acceptor_variant&splice_region_variant&intron_variant	Uncertain_significance
ly6874	SYNE1	c.19087G>A	p.Glu6363Lys	missense_variant	Uncertain_significance
ly6874	SLC6A20	c.1477C>A	p.Leu493Ile	missense_variant	Uncertain_significance
ly6874	AGXT	c.1111G>A	p.Glu371Lys	missense_variant	Uncertain_significance
ly6874	JUP	c.2207C>A	p.Pro736Gln	missense_variant	Uncertain_significance
ly6874	PHKB	c.491A>G	p.Tyr164Cys	missense_variant	Uncertain_significance
ly6874	LRRK2	c.3974G>A	p.Arg1325Gln	missense_variant	Uncertain_significance
ly6874	RBM20	c.3595G>T	p.Glu1199*	stop_gained	Uncertain_significance
ly6874	PALB2	c.1707dupA	p.Glu570fs	frameshift_variant	Uncertain_significance
ly6875	CSF1R	c.1420G>T	p.Val474Phe	missense_variant	Uncertain_significance
ly6875	BARD1	c.2294A>G	p.Asp765Gly	missense_variant	Uncertain_significance
ly6875	TTN	c.11855G>T	p.Gly3952Val	missense_variant	Uncertain_significance

ly6875	GAA	c.2275G>T	p.Gly759Trp	missense_variant	Uncertain_significance
ly6875	GPR179	c.6335C>T	p.Ala2112Val	missense_variant	Uncertain_significance
ly6875	CDK4	c.736C>A	p.Arg246Ser	missense_variant	Uncertain_significance
ly6875	DMD	c.5326-3dupT		splice_acceptor_variant&splice_region_variant&intron_variant	Uncertain_significance
ly6876	C5orf42	c.7099A>G	p.Lys2367Glu	missense_variant	Uncertain_significance
ly6876	MSH2	c.1945G>C	p.Ala649Pro	missense_variant	Uncertain_significance
ly6876	TTN	c.39820C>T	p.Pro13274Ser	missense_variant&splice_region_variant	Uncertain_significance
ly6876	ERCC2	c.691G>A	p.Val231Met	missense_variant	Uncertain_significance
ly6876	MLH3	c.2021dupA	p.Asn674fs	frameshift_variant	Uncertain_significance
ly6876	POLE	c.968C>G	p.Thr323Ser	missense_variant	Uncertain_significance
ly6876	NDUFV1	c.563G>T	p.Gly188Val	missense_variant	Uncertain_significance
ly6876	KCNQ1	c.1128+5G>A		splice_donor_variant&splice_region_variant&intron_variant	Uncertain_significance
ly6877	RECQL4	c.380C>T	p.Pro127Leu	missense_variant	Uncertain_significance
ly6877	PLEC	c.9226C>T	p.Arg3076Trp	missense_variant	Uncertain_significance
ly6877	AKAP9	c.11246G>T	p.Gly3749Val	missense_variant	Uncertain_significance
ly6877	CHRM2	c.703C>A	p.Leu235Met	missense_variant	Uncertain_significance
ly6877	RINT1	c.1187A>G	p.Asn396Ser	missense_variant	Uncertain_significance
ly6877	JUP	c.1873G>T	p.Gly625Trp	missense_variant	Uncertain_significance
ly6877	ERCC5	c.670G>T	p.Glu224*	stop_gained&splice_region_variant	Uncertain_significance

ly6877	C2orf71	c.2965C>G	p.Pro989Ala	missense_variant	Uncertain_significance
ly6878	RIMS1	c.165-5C>G		splice_acceptor_variant&splice_region_variant&intron_variant	Uncertain_significance
ly6878	CPOX	c.651A>T	p.Glu217Asp	missense_variant	Uncertain_significance
ly6878	RFT1	c.136G>A	p.Val46Ile	missense_variant	Uncertain_significance
ly6878	SCN5A	c.1840C>A	p.Pro614Thr	missense_variant	Uncertain_significance
ly6878	CREBBP	c.6743_6745delAGC	p.Gln2248del	disruptive_inframe_deletion	Uncertain_significance
ly6878	FANCI	c.1741G>A	p.Glu581Lys	missense_variant	Uncertain_significance
ly6878	CDHR1	c.2027T>A	p.Ile676Asn	missense_variant	Uncertain_significance
ly6879	MMACHC	c.334C>T		protein_protein_contact	Uncertain_significance
ly6879	WRAP53	c.395C>T	p.Thr132Ile	missense_variant	Uncertain_significance
ly6879	FANCI	c.3400A>G	p.Ile1134Val	missense_variant	Uncertain_significance
ly6879	GNPTAB	c.3710G>A	p.Arg1237Gln	missense_variant	Uncertain_significance
ly6879	BARD1	c.233G>A	p.Cys78Tyr	missense_variant	Uncertain_significance
ly6880	DNAH11	c.9890C>G	p.Ser3297Cys	missense_variant	Uncertain_significance
ly6880	IMPG2	c.1582A>G	p.Ile528Val	missense_variant	Uncertain_significance
ly6881	B4GALT1	c.517G>A		protein_protein_contact	Uncertain_significance
ly6881	FAM161A	c.916C>T	p.Arg306Trp	missense_variant	Uncertain_significance
ly6881	PCNT	c.4605G>T	p.Lys1535Asn	missense_variant	Uncertain_significance
ly6881	KL	c.511C>A	p.Arg171Ser	missense_variant	Uncertain_significance
ly6882	ALG13	c.1798A>G	p.Met600Val	missense_variant	Uncertain_significance
ly6883	NBN	c.1912T>C	p.Ser638Pro	missense_variant&splice_region_variant	Uncertain_significance

ly6883	CYP4F22	c.736C>T	p.Arg246Cys	missense_variant	Uncertain_significance
ly6883	ALPK3	c.3280G>C	p.Gly1094Arg	missense_variant	Uncertain_significance
ly6883	POLE	c.6539C>T	p.Ala2180Val	missense_variant	Uncertain_significance
ly6883	FAS	c.444-5dupT		splice_acceptor_variant&splice_region_variant&intron_variant	Uncertain_significance
ly6884	CHRNA2	c.1015G>A	p.Val339Ile	missense_variant	Uncertain_significance
ly6884	C5orf42	c.7298T>C	p.Leu2433Pro	missense_variant	Uncertain_significance
ly6884	KIT	c.1847C>T	p.Ala616Val	missense_variant	Uncertain_significance
ly6884	SCN1A	c.853G>T	p.Ala285Ser	missense_variant	Uncertain_significance
ly6884	SNTA1	c.1181G>A	p.Arg394His	missense_variant	Uncertain_significance
ly6884	PEX10	c.956C>T	p.Ala319Val	missense_variant	Uncertain_significance
ly6884	SMCHD1	c.5411T>C	p.Ile1804Thr	missense_variant	Uncertain_significance
ly6885	CSTB	c.146C>T	p.Ala49Val	missense_variant	Uncertain_significance
ly6885	NPHP4	c.2198G>A	p.Gly733Asp	missense_variant	Uncertain_significance
ly6885	LAMC2	c.1147A>G	p.Ile383Val	missense_variant	Uncertain_significance
ly6885	SCN4A	c.1120G>A	p.Glu374Lys	missense_variant	Uncertain_significance
ly6885	GPR56	c.761G>A	p.Arg254Gln	missense_variant	Uncertain_significance
ly6885	SYNE2	c.13461G>A	p.Met4487Ile	missense_variant	Uncertain_significance
ly6885	KRT83	c.910A>T	p.Ser304Cys	missense_variant	Uncertain_significance
ly6885	MRE11A	c.2092_2094delGAT	p.Asp698del	inframe_deletion	Uncertain_significance
ly6886	CNTNAP2	c.2047G>T	p.Glu683*	stop_gained	Uncertain_significance
ly6886	MET	c.4199G>T	p.Arg1400Leu	missense_variant	Uncertain_significance
ly6886	DSP	c.4072_4074delGAG	p.Glu1358del	inframe_deletion	Uncertain_significance

ly6886	TTN	c.81527G>A	p.Arg27176His	missense_variant	Uncertain_significance
ly6886	LAMB3	c.1807C>T	p.Arg603Cys	missense_variant	Uncertain_significance
ly6886	SDHC	c.40C>A	p.Leu14Ile	missense_variant	Uncertain_significance
ly6886	RYR1	c.9523C>A	p.Leu3175Met	missense_variant	Uncertain_significance
ly6886	JUP	c.1909C>A		protein_protein_contact	Uncertain_significance
ly6886	POLG	c.1832C>A	p.Pro611His	missense_variant	Uncertain_significance
ly6886	OCA2	c.1453G>T	p.Gly485Trp	missense_variant	Uncertain_significance
ly6886	NPC2	c.271G>A	p.Asp91Asn	missense_variant	Uncertain_significance
ly6886	VWF	c.4123C>A	p.Pro1375Thr	missense_variant	Uncertain_significance
ly6886	CTSC	c.565A>G	p.Thr189Ala	missense_variant	Uncertain_significance
ly6886	KIAA1279	c.1083delA	p.Ala362fs	frameshift_variant	Uncertain_significance
ly6887	LAMA4	c.307C>T	p.Arg103Trp	missense_variant	Uncertain_significance
ly6887	DYSF	c.6100A>C	p.Ser2034Arg	missense_variant	Uncertain_significance
ly6887	NPHP4	c.2531C>T	p.Pro844Leu	missense_variant	Uncertain_significance
ly6887	ARHGEF15	c.1810C>T	p.Arg604Cys	missense_variant	Uncertain_significance
ly6887	SPRED1	c.881A>T	p.Tyr294Phe	missense_variant	Uncertain_significance
ly6888	FREM1	c.2788G>T	p.Val930Leu	missense_variant	Uncertain_significance
ly6888	CHIT1	c.767C>T	p.Pro256Leu	missense_variant	Uncertain_significance
ly6888	PNPO	c.698G>T	p.Arg233Leu	missense_variant	Uncertain_significance
ly6888	KIF7	c.2237G>T	p.Arg746Leu	missense_variant	Uncertain_significance
ly6888	SYNE2	c.16730G>T	p.Arg5577Leu	missense_variant	Uncertain_significance
ly6888	MSH6	c.2045C>A	p.Ser682Tyr	missense_variant	Uncertain_significance
ly6890	FRAS1	c.6569C>T	p.Ser2190Phe	missense_variant	Uncertain_significance
ly6890	KIT	c.2263G>A	p.Ala755Thr	missense_variant	Uncertain_significance

ly6891	FLNB	c.5135C>G	p.Pro1712Arg	missense_variant	Uncertain_significance
ly6891	FLNB	c.1748-5C>G		splice_acceptor_variant&splice_region_variant&intron_variant	Uncertain_significance
ly6891	TTN	c.89924C>T	p.Ala29975Val	missense_variant	Uncertain_significance
ly6891	ABCC9	c.826G>A	p.Ala276Thr	missense_variant	Uncertain_significance
ly6892	XRCC2	c.789_790delCA	p.Asn263fs	frameshift_variant	Uncertain_significance
ly6892	NRXN1	c.4180A>T	p.Thr1394Ser	missense_variant	Uncertain_significance
ly6893	FRAS1	c.11902A>G	p.Arg3968Gly	missense_variant	Uncertain_significance
ly6893	DYSF	c.2257C>A	p.His753Asn	missense_variant	Uncertain_significance
